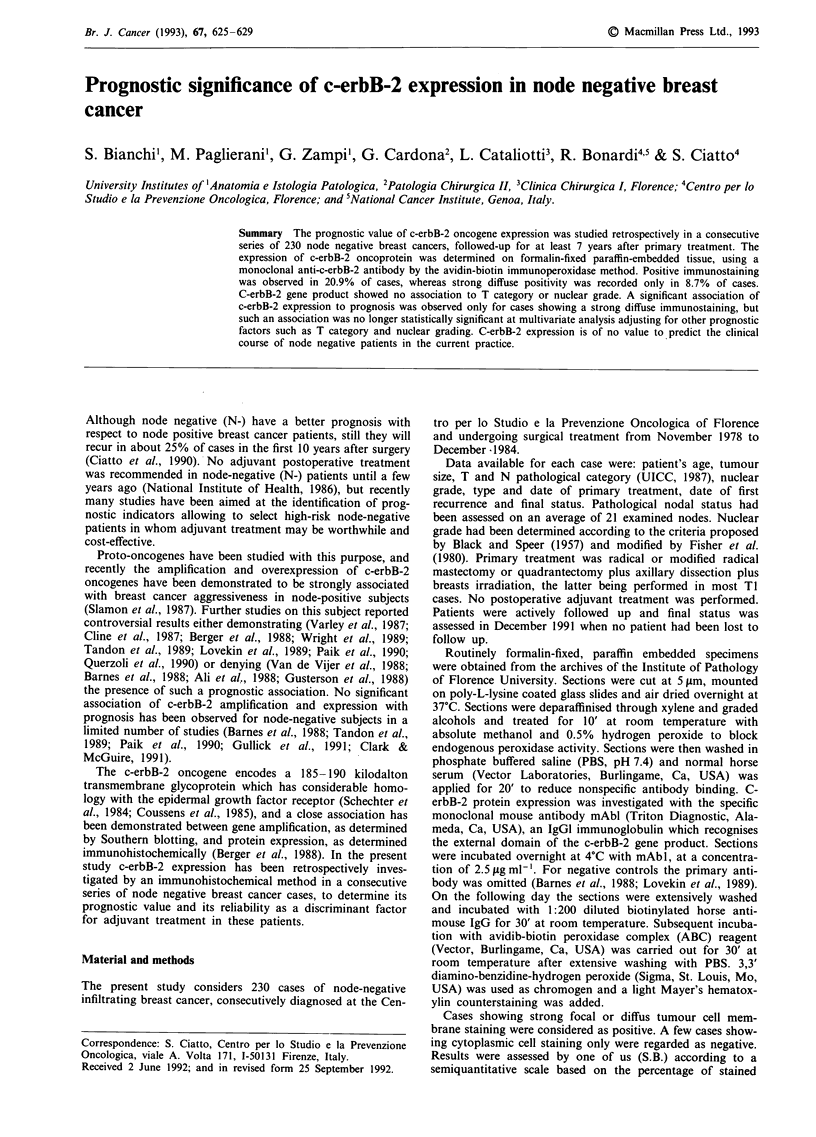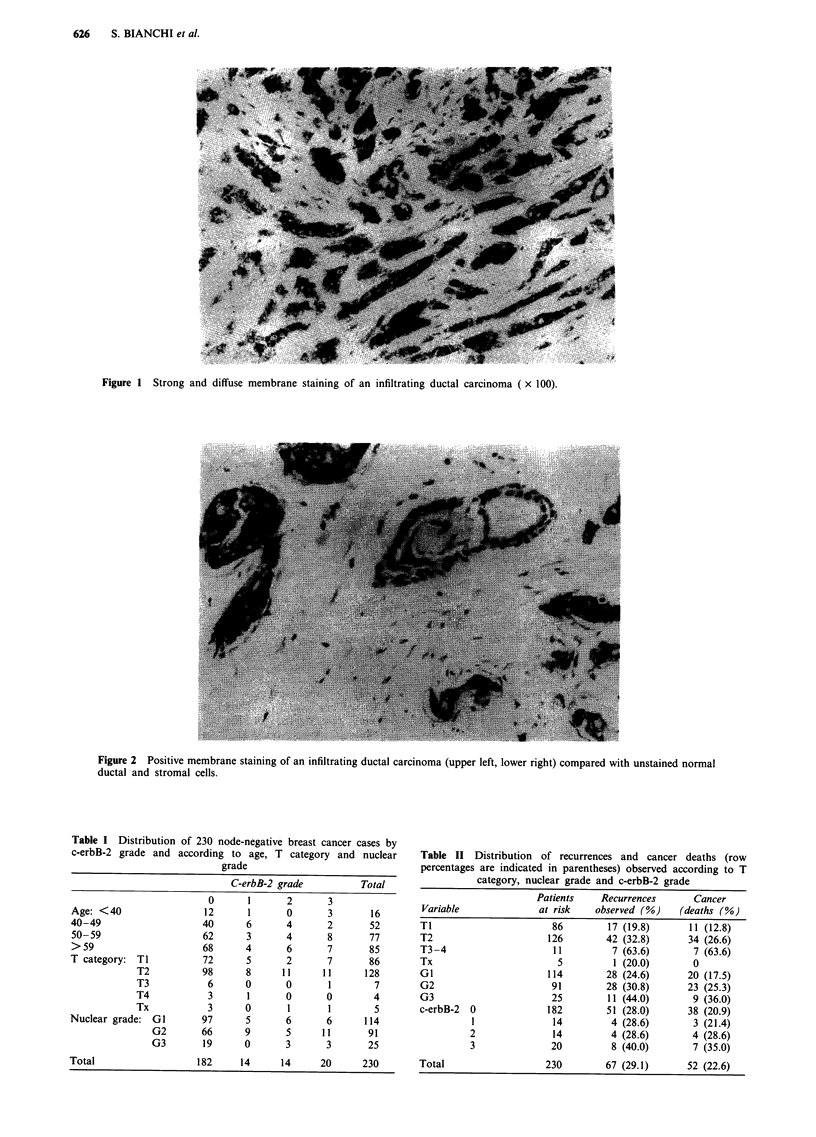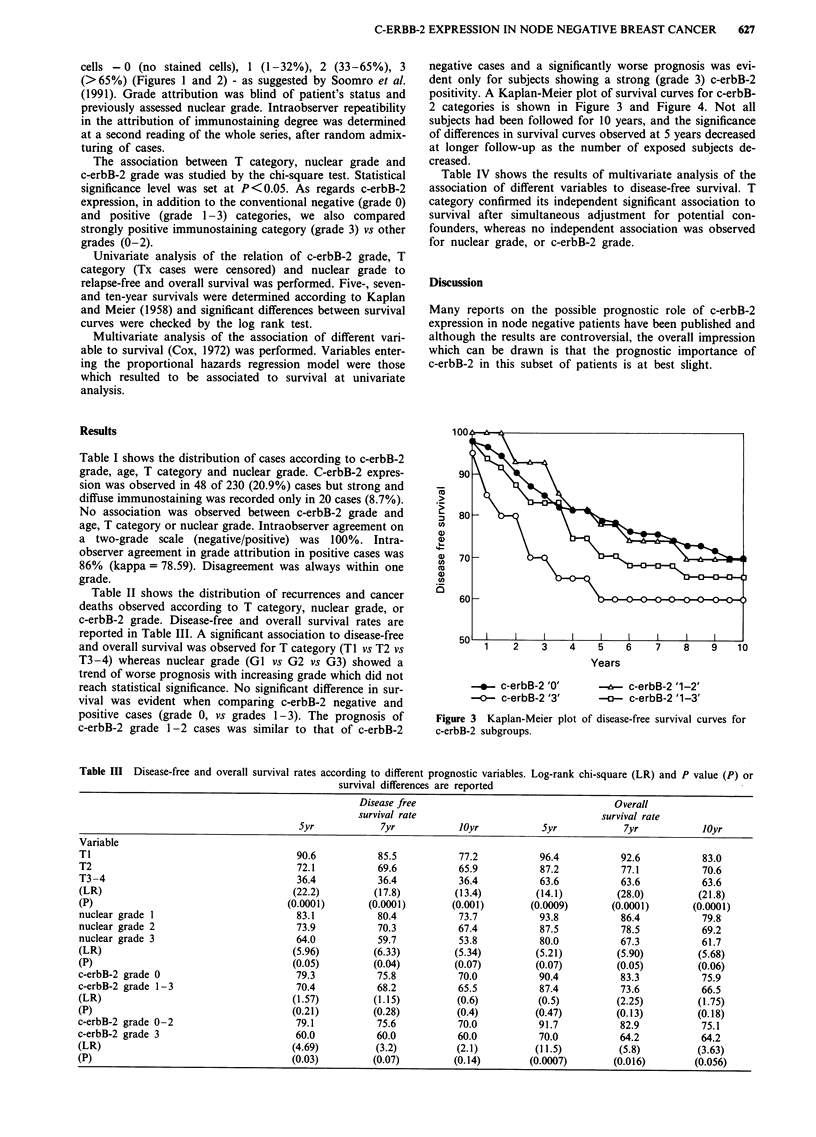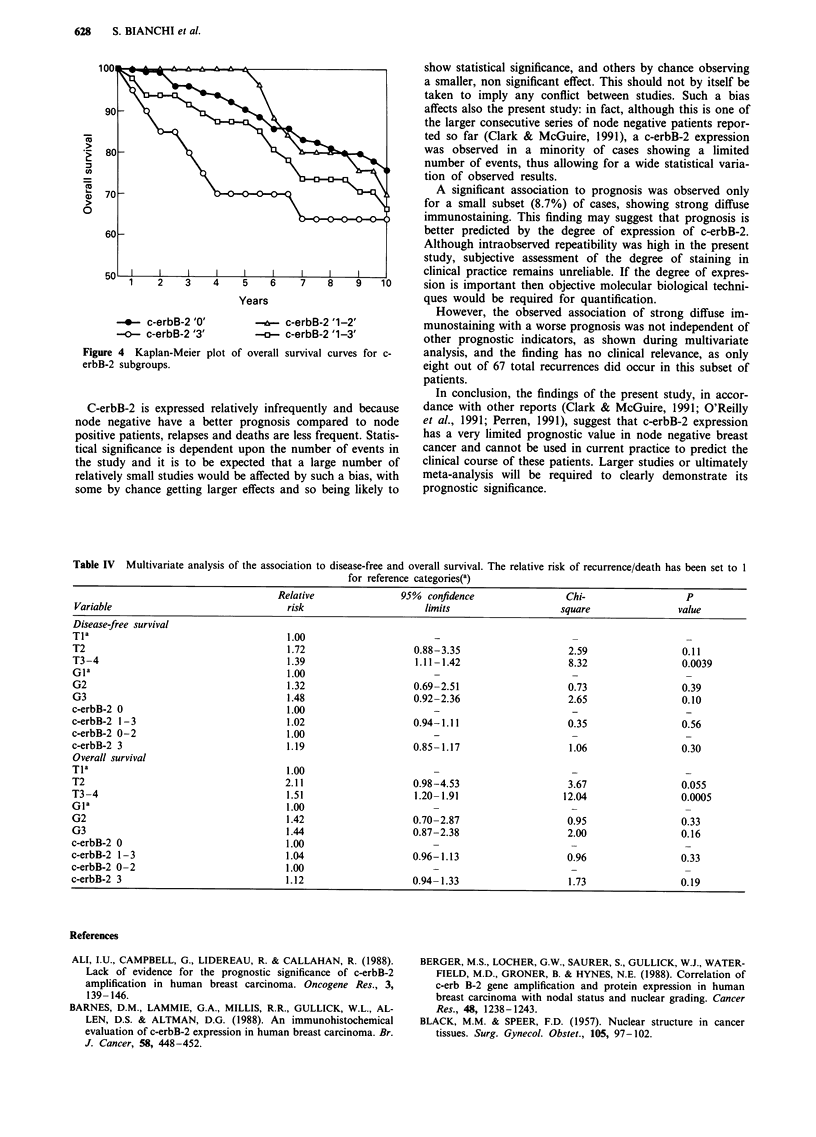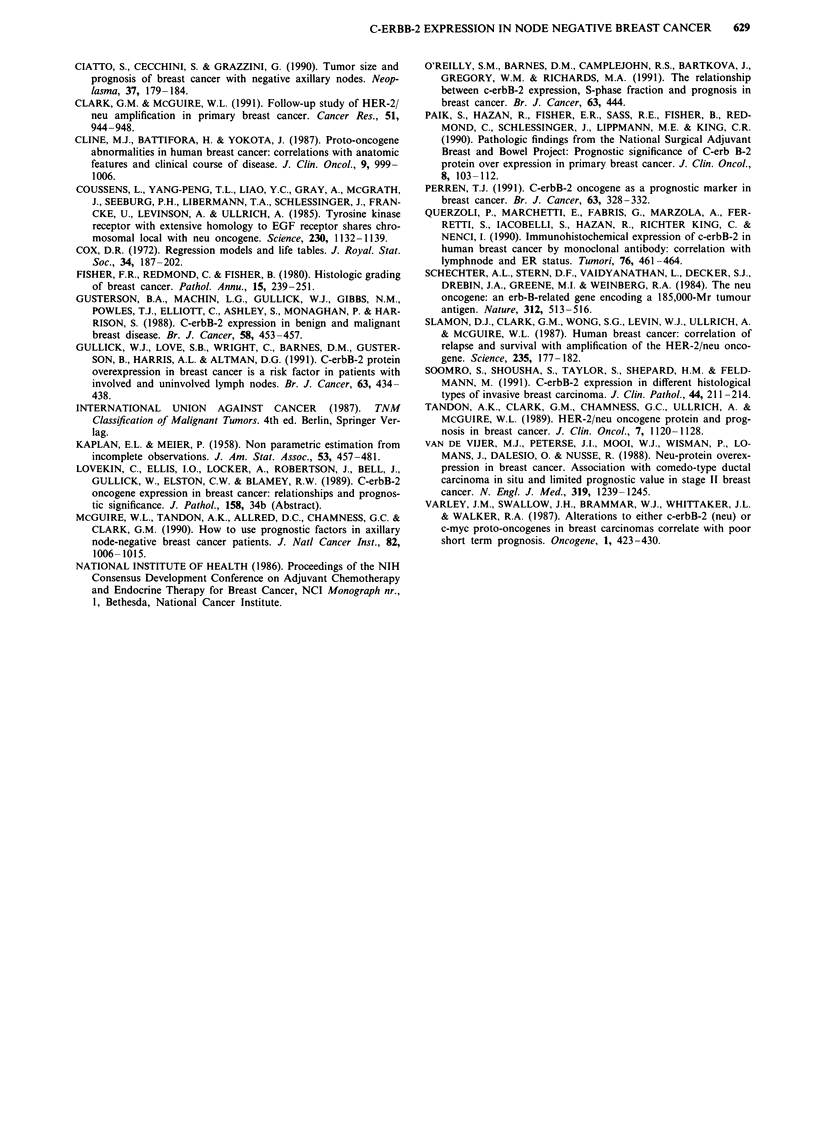# Prognostic significance of c-erbB-2 expression in node negative breast cancer.

**DOI:** 10.1038/bjc.1993.114

**Published:** 1993-03

**Authors:** S. Bianchi, M. Paglierani, G. Zampi, G. Cardona, L. Cataliotti, R. Bonardi, S. Ciatto

**Affiliations:** University Institute of Anatomia e Istologia Patologica, Florence, Italy.

## Abstract

**Images:**


Br. J. Cancer (1993), 67, 625-629 © Macmillan Press Ltd., 1993

### Prognostic significance of c-erbB-2 expression in node negative breast cancer

S. Bianchi[1], M. Paglierani[1], G. Zampi[1], G. Cardona[2], L. Cataliotti[3], R. Bonardi[4,5] & S. Ciatto[4]

*University Institutes of [1]Anatomia e Istologia Patologica, [2]Patologia Chirurgica II, [3]Clinica Chirurgica I, Florence; [4]Centro per lo Studio e la Prevenzione Oncologica, Florence; and [5]National Cancer Institute, Genoa, Italy.*

**Summary** The prognostic value of c-erbB-2 oncogene expression was studied retrospectively in a consecutive series of 230 node negative breast cancers, followed-up for at least 7 years after primary treatment. The expression of c-erbB-2 oncoprotein was determined on formalin-fixed paraffin-embedded tissue, using a monoclonal anti-c-erbB-2 antibody by the avidin-biotin immunoperoxidase method. Positive immunostaining was observed in 20.9% of cases, whereas strong diffuse positivity was recorded only in 8.7% of cases. C-erbB-2 gene product showed no association to T category or nuclear grade. A significant association of c-erbB-2 expression to prognosis was observed only for cases showing a strong diffuse immunostaining, but such an association was no longer statistically significant at multivariate analysis adjusting for other prognostic factors such as T category and nuclear grading. C-erbB-2 expression is of no value to predict the clinical course of node negative patients in the current practice.

Although node negative (N-) have a better prognosis with respect to node positive breast cancer patients, still they will recur in about 25% of cases in the first 10 years after surgery (Ciatto *et al.*, 1990). No adjuvant postoperative treatment was recommended in node-negative (N-) patients until a few years ago (National Institute of Health, 1986), but recently many studies have been aimed at the identification of prognostic indicators allowing to select high-risk node-negative patients in whom adjuvant treatment may be worthwhile and cost-effective.

Proto-oncogenes have been studied with this purpose, and recently the amplification and overexpression of c-erbB-2 oncogenes have been demonstrated to be strongly associated with breast cancer aggressiveness in node-positive subjects (Slamon *et al.*, 1987). Further studies on this subject reported controversial results either demonstrating (Varley *et al.*, 1987; Cline *et al.*, 1987; Berger *et al.*, 1988; Wright *et al.*, 1989; Tandon *et al.*, 1989; Lovekin *et al.*, 1989; Paik *et al.*, 1990; Querzoli *et al.*, 1990) or denying (Van de Vijer *et al.*, 1988; Barnes *et al.*, 1988; Ali *et al.*, 1988; Gusterson *et al.*, 1988) the presence of such a prognostic association. No significant association of c-erbB-2 amplification and expression with prognosis has been observed for node-negative subjects in a limited number of studies (Barnes *et al.*, 1988; Tandon *et al.*, 1989; Paik *et al.*, 1990; Gullick *et al.*, 1991; Clark & McGuire, 1991).

The c-erbB-2 oncogene encodes a 185-190 kilodalton transmembrane glycoprotein which has considerable homology with the epidermal growth factor receptor (Schechter *et al.*, 1984; Coussens *et al.*, 1985), and a close association has been demonstrated between gene amplification, as determined by Southern blotting, and protein expression, as determined immunohistochemically (Berger *et al.*, 1988). In the present study c-erbB-2 expression has been retrospectively investigated by an immunohistochemical method in a consecutive series of node negative breast cancer cases, to determine its prognostic value and its reliability as a discriminant factor for adjuvant treatment in these patients.

### Material and methods

The present study considers 230 cases of node-negative infiltrating breast cancer, consecutively diagnosed at the Centro per lo Studio e la Prevenzione Oncologica of Florence and undergoing surgical treatment from November 1978 to December 1984.

Data available for each case were: patient's age, tumour size, T and N pathological category (UICC, 1987), nuclear grade, type and date of primary treatment, date of first recurrence and final status. Pathological nodal status had been assessed on an average of 21 examined nodes. Nuclear grade had been determined according to the criteria proposed by Black and Speer (1957) and modified by Fisher *et al.* (1980). Primary treatment was radical or modified radical mastectomy or quadrantectomy plus axillary dissection plus breasts irradiation, the latter being performed in most T1 cases. No postoperative adjuvant treatment was performed. Patients were actively followed up and final status was assessed in December 1991 when no patient had been lost to follow up.

Routinely formalin-fixed, paraffin embedded specimens were obtained from the archives of the Institute of Pathology of Florence University. Sections were cut at 5 μm, mounted on poly-L-lysine coated glass slides and air dried overnight at 37°C. Sections were deparaffinised through xylene and graded alcohols and treated for 10′ at room temperature with absolute methanol and 0.5% hydrogen peroxide to block endogenous peroxidase activity. Sections were then washed in phosphate buffered saline (PBS, pH 7.4) and normal horse serum (Vector Laboratories, Burlingame, Ca, USA) was applied for 20′ to reduce nonspecific antibody binding. C-erbB-2 protein expression was investigated with the specific monoclonal mouse antibody mAbl (Triton Diagnostic, Alameda, Ca, USA), an IgGl immunoglobulin which recognises the external domain of the c-erbB-2 gene product. Sections were incubated overnight at 4°C with mAb1, at a concentration of 2.5 $\mu g\, ml^{-1}$. For negative controls the primary antibody was omitted (Barnes *et al.*, 1988; Lovekin *et al.*, 1989). On the following day the sections were extensively washed and incubated with 1:200 diluted biotinylated horse anti-mouse IgG for 30′ at room temperature. Subsequent incubation with avidib-biotin peroxidase complex (ABC) reagent (Vector, Burlingame, Ca, USA) was carried out for 30′ at room temperature after extensive washing with PBS. 3,3′ diamino-benzidine-hydrogen peroxide (Sigma, St. Louis, Mo, USA) was used as chromogen and a light Mayer's hematoxylin counterstaining was added.

Cases showing strong focal or diffus tumour cell membrane staining were considered as positive. A few cases showing cytoplasmic cell staining only were regarded as negative. Results were assessed by one of us (S.B.) according to a semiquantitative scale based on the percentage of stained

Correspondence: S. Ciatto, Centro per lo Studio e la Prevenzione Oncologica, viale A. Volta 171, I-50131 Firenze, Italy.
Received 2 June 1992; and in revised form 25 September 1992.

**Figure 1** Strong and diffuse membrane staining of an infiltrating ductal carcinoma ( × 100).

**Figure 2** Positive membrane staining of an infiltrating ductal carcinoma (upper left, lower right) compared with unstained normal ductal and stromal cells.

**Table I** Distribution of 230 node-negative breast cancer cases by c-erbB-2 grade and according to age, T category and nuclear grade

| | *C-erbB-2 grade* | | | | *Total* |
|---|---|---|---|---|---|
| | 0 | 1 | 2 | 3 | |
| Age: <40 | 12 | 1 | 0 | 3 | 16 |
| 40–49 | 40 | 6 | 4 | 2 | 52 |
| 50–59 | 62 | 3 | 4 | 8 | 77 |
| >59 | 68 | 4 | 6 | 7 | 85 |
| T category: T1 | 72 | 5 | 2 | 7 | 86 |
| T2 | 98 | 8 | 11 | 11 | 128 |
| T3 | 6 | 0 | 0 | 1 | 7 |
| T4 | 3 | 1 | 0 | 0 | 4 |
| Tx | 3 | 0 | 1 | 1 | 5 |
| Nuclear grade: G1 | 97 | 5 | 6 | 6 | 114 |
| G2 | 66 | 9 | 5 | 11 | 91 |
| G3 | 19 | 0 | 3 | 3 | 25 |
| Total | 182 | 14 | 14 | 20 | 230 |

**Table II** Distribution of recurrences and cancer deaths (row percentages are indicated in parentheses) observed according to T category, nuclear grade and c-erbB-2 grade

| *Variable* | *Patients at risk* | *Recurrences observed (%)* | *Cancer (deaths (%)* |
|---|---|---|---|
| T1 | 86 | 17 (19.8) | 11 (12.8) |
| T2 | 126 | 42 (32.8) | 34 (26.6) |
| T3–4 | 11 | 7 (63.6) | 7 (63.6) |
| Tx | 5 | 1 (20.0) | 0 |
| G1 | 114 | 28 (24.6) | 20 (17.5) |
| G2 | 91 | 28 (30.8) | 23 (25.3) |
| G3 | 25 | 11 (44.0) | 9 (36.0) |
| c-erbB-2 0 | 182 | 51 (28.0) | 38 (20.9) |
| 1 | 14 | 4 (28.6) | 3 (21.4) |
| 2 | 14 | 4 (28.6) | 4 (28.6) |
| 3 | 20 | 8 (40.0) | 7 (35.0) |
| Total | 230 | 67 (29.1) | 52 (22.6) |

cells – 0 (no stained cells), 1 (1–32%), 2 (33–65%), 3 (>65%) (Figures 1 and 2) - as suggested by Soomro *et al.* (1991). Grade attribution was blind of patient's status and previously assessed nuclear grade. Intraobserver repeatibility in the attribution of immunostaining degree was determined at a second reading of the whole series, after random admixturing of cases.

The association between T category, nuclear grade and c-erbB-2 grade was studied by the chi-square test. Statistical significance level was set at $P<0.05$. As regards c-erbB-2 expression, in addition to the conventional negative (grade 0) and positive (grade 1–3) categories, we also compared strongly positive immunostaining category (grade 3) *vs* other grades (0–2).

Univariate analysis of the relation of c-erbB-2 grade, T category (Tx cases were censored) and nuclear grade to relapse-free and overall survival was performed. Five-, seven- and ten-year survivals were determined according to Kaplan and Meier (1958) and significant differences between survival curves were checked by the log rank test.

Multivariate analysis of the association of different variable to survival (Cox, 1972) was performed. Variables entering the proportional hazards regression model were those which resulted to be associated to survival at univariate analysis.

### Results

Table I shows the distribution of cases according to c-erbB-2 grade, age, T category and nuclear grade. C-erbB-2 expression was observed in 48 of 230 (20.9%) cases but strong and diffuse immunostaining was recorded only in 20 cases (8.7%). No association was observed between c-erbB-2 grade and age, T category or nuclear grade. Intraobserver agreement on a two-grade scale (negative/positive) was 100%. Intraobserver agreement in grade attribution in positive cases was 86% (kappa = 78.59). Disagreement was always within one grade.

Table II shows the distribution of recurrences and cancer deaths observed according to T category, nuclear grade, or c-erbB-2 grade. Disease-free and overall survival rates are reported in Table III. A significant association to disease-free and overall survival was observed for T category (T1 *vs* T2 *vs* T3–4) whereas nuclear grade (G1 *vs* G2 *vs* G3) showed a trend of worse prognosis with increasing grade which did not reach statistical significance. No significant difference in survival was evident when comparing c-erbB-2 negative and positive cases (grade 0, *vs* grades 1–3). The prognosis of c-erbB-2 grade 1–2 cases was similar to that of c-erbB-2 negative cases and a significantly worse prognosis was evident only for subjects showing a strong (grade 3) c-erbB-2 positivity. A Kaplan-Meier plot of survival curves for c-erbB-2 categories is shown in Figure 3 and Figure 4. Not all subjects had been followed for 10 years, and the significance of differences in survival curves observed at 5 years decreased at longer follow-up as the number of exposed subjects decreased.

Table IV shows the results of multivariate analysis of the association of different variables to disease-free survival. T category confirmed its independent significant association to survival after simultaneous adjustment for potential confounders, whereas no independent association was observed for nuclear grade, or c-erbB-2 grade.

### Discussion

Many reports on the possible prognostic role of c-erbB-2 expression in node negative patients have been published and although the results are controversial, the overall impression which can be drawn is that the prognostic importance of c-erbB-2 in this subset of patients is at best slight.

**Figure 3** Kaplan-Meier plot of disease-free survival curves for c-erbB-2 subgroups.

**Table III** Disease-free and overall survival rates according to different prognostic variables. Log-rank chi-square (LR) and *P* value (*P*) or survival differences are reported

| | Disease free survival rate | | | Overall survival rate | | |
|---|---|---|---|---|---|---|
| | *5yr* | *7yr* | *10yr* | *5yr* | *7yr* | *10yr* |
| Variable | | | | | | |
| T1 | 90.6 | 85.5 | 77.2 | 96.4 | 92.6 | 83.0 |
| T2 | 72.1 | 69.6 | 65.9 | 87.2 | 77.1 | 70.6 |
| T3–4 | 36.4 | 36.4 | 36.4 | 63.6 | 63.6 | 63.6 |
| (LR) | (22.2) | (17.8) | (13.4) | (14.1) | (28.0) | (21.8) |
| (P) | (0.0001) | (0.0001) | (0.001) | (0.0009) | (0.0001) | (0.0001) |
| nuclear grade 1 | 83.1 | 80.4 | 73.7 | 93.8 | 86.4 | 79.8 |
| nuclear grade 2 | 73.9 | 70.3 | 67.4 | 87.5 | 78.5 | 69.2 |
| nuclear grade 3 | 64.0 | 59.7 | 53.8 | 80.0 | 67.3 | 61.7 |
| (LR) | (5.96) | (6.33) | (5.34) | (5.21) | (5.90) | (5.68) |
| (P) | (0.05) | (0.04) | (0.07) | (0.07) | (0.05) | (0.06) |
| c-erbB-2 grade 0 | 79.3 | 75.8 | 70.0 | 90.4 | 83.3 | 75.9 |
| c-erbB-2 grade 1–3 | 70.4 | 68.2 | 65.5 | 87.4 | 73.6 | 66.5 |
| (LR) | (1.57) | (1.15) | (0.6) | (0.5) | (2.25) | (1.75) |
| (P) | (0.21) | (0.28) | (0.4) | (0.47) | (0.13) | (0.18) |
| c-erbB-2 grade 0–2 | 79.1 | 75.6 | 70.0 | 91.7 | 82.9 | 75.1 |
| c-erbB-2 grade 3 | 60.0 | 60.0 | 60.0 | 70.0 | 64.2 | 64.2 |
| (LR) | (4.69) | (3.2) | (2.1) | (11.5) | (5.8) | (3.63) |
| (P) | (0.03) | (0.07) | (0.14) | (0.0007) | (0.016) | (0.056) |

**Figure 4** Kaplan-Meier plot of overall survival curves for c-erbB-2 subgroups.

C-erbB-2 is expressed relatively infrequently and because node negative have a better prognosis compared to node positive patients, relapses and deaths are less frequent. Statistical significance is dependent upon the number of events in the study and it is to be expected that a large number of relatively small studies would be affected by such a bias, with some by chance getting larger effects and so being likely to show statistical significance, and others by chance observing a smaller, non significant effect. This should not by itself be taken to imply any conflict between studies. Such a bias affects also the present study: in fact, although this is one of the larger consecutive series of node negative patients reported so far (Clark & McGuire, 1991), a c-erbB-2 expression was observed in a minority of cases showing a limited number of events, thus allowing for a wide statistical variation of observed results.

A significant association to prognosis was observed only for a small subset (8.7%) of cases, showing strong diffuse immunostaining. This finding may suggest that prognosis is better predicted by the degree of expression of c-erbB-2. Although intraobserved repeatibility was high in the present study, subjective assessment of the degree of staining in clinical practice remains unreliable. If the degree of expression is important then objective molecular biological techniques would be required for quantification.

However, the observed association of strong diffuse immunostaining with a worse prognosis was not independent of other prognostic indicators, as shown during multivariate analysis, and the finding has no clinical relevance, as only eight out of 67 total recurrences did occur in this subset of patients.

In conclusion, the findings of the present study, in accordance with other reports (Clark & McGuire, 1991; O'Reilly *et al.*, 1991; Perren, 1991), suggest that c-erbB-2 expression has a very limited prognostic value in node negative breast cancer and cannot be used in current practice to predict the clinical course of these patients. Larger studies or ultimately meta-analysis will be required to clearly demonstrate its prognostic significance.

**Table IV** Multivariate analysis of the association to disease-free and overall survival. The relative risk of recurrence/death has been set to 1 for reference categories([a])

| *Variable* | *Relative risk* | *95% confidence limits* | *Chi-square* | *P value* |
|---|---|---|---|---|
| *Disease-free survival* | | | | |
| T1[a] | 1.00 | – | – | – |
| T2 | 1.72 | 0.88–3.35 | 2.59 | 0.11 |
| T3–4 | 1.39 | 1.11–1.42 | 8.32 | 0.0039 |
| G1[a] | 1.00 | – | – | – |
| G2 | 1.32 | 0.69–2.51 | 0.73 | 0.39 |
| G3 | 1.48 | 0.92–2.36 | 2.65 | 0.10 |
| c-erbB-2 0 | 1.00 | – | – | – |
| c-erbB-2 1–3 | 1.02 | 0.94–1.11 | 0.35 | 0.56 |
| c-erbB-2 0–2 | 1.00 | – | – | – |
| c-erbB-2 3 | 1.19 | 0.85–1.17 | 1.06 | 0.30 |
| *Overall survival* | | | | |
| T1[a] | 1.00 | – | – | – |
| T2 | 2.11 | 0.98–4.53 | 3.67 | 0.055 |
| T3–4 | 1.51 | 1.20–1.91 | 12.04 | 0.0005 |
| G1[a] | 1.00 | – | – | – |
| G2 | 1.42 | 0.70–2.87 | 0.95 | 0.33 |
| G3 | 1.44 | 0.87–2.38 | 2.00 | 0.16 |
| c-erbB-2 0 | 1.00 | – | – | – |
| c-erbB-2 1–3 | 1.04 | 0.96–1.13 | 0.96 | 0.33 |
| c-erbB-2 0–2 | 1.00 | – | – | – |
| c-erbB-2 3 | 1.12 | 0.94–1.33 | 1.73 | 0.19 |

### References

ALI, I.U., CAMPBELL, G., LIDEREAU, R. & CALLAHAN, R. (1988). Lack of evidence for the prognostic significance of c-erbB-2 amplification in human breast carcinoma. *Oncogene Res.*, **3**, 139–146.

BARNES, D.M., LAMMIE, G.A., MILLIS, R.R., GULLICK, W.L., ALLEN, D.S. & ALTMAN, D.G. (1988). An immunohistochemical evaluation of c-erbB-2 expression in human breast carcinoma. *Br. J. Cancer*, **58**, 448–452.

BERGER, M.S., LOCHER, G.W., SAURER, S., GULLICK, W.J., WATERFIELD, M.D., GRONER, B. & HYNES, N.E. (1988). Correlation of c-erb B-2 gene amplification and protein expression in human breast carcinoma with nodal status and nuclear grading. *Cancer Res.*, **48**, 1238–1243.

BLACK, M.M. & SPEER, F.D. (1957). Nuclear structure in cancer tissues. *Surg. Gynecol. Obstet.*, **105**, 97–102.

CIATTO, S., CECCHINI, S. & GRAZZINI, G. (1990). Tumor size and prognosis of breast cancer with negative axillary nodes. *Neoplasma*, **37**, 179–184.

CLARK, G.M. & MCGUIRE, W.L. (1991). Follow-up study of HER-2/neu amplification in primary breast cancer. *Cancer Res.*, **51**, 944–948.

CLINE, M.J., BATTIFORA, H. & YOKOTA, J. (1987). Proto-oncogene abnormalities in human breast cancer: correlations with anatomic features and clinical course of disease. *J. Clin. Oncol.*, **9**, 999–1006.

COUSSENS, L., YANG-PENG, T.L., LIAO, Y.C., GRAY, A., MCGRATH, J., SEEBURG, P.H., LIBERMANN, T.A., SCHLESSINGER, J., FRANCKE, U., LEVINSON, A. & ULLRICH, A. (1985). Tyrosine kinase receptor with extensive homology to EGF receptor shares chromosomal local with neu oncogene. *Science*, **230**, 1132–1139.

COX, D.R. (1972). Regression models and life tables. *J. Royal. Stat. Soc.*, **34**, 187–202.

FISHER, F.R., REDMOND, C. & FISHER, B. (1980). Histologic grading of breast cancer. *Pathol. Annu.*, **15**, 239–251.

GUSTERSON, B.A., MACHIN, L.G., GULLICK, W.J., GIBBS, N.M., POWLES, T.J., ELLIOTT, C., ASHLEY, S., MONAGHAN, P. & HARRISON, S. (1988). C-erbB-2 expression in benign and malignant breast disease. *Br. J. Cancer*, **58**, 453–457.

GULLICK, W.J., LOVE, S.B., WRIGHT, C., BARNES, D.M., GUSTERSON, B., HARRIS, A.L. & ALTMAN, D.G. (1991). C-erbB-2 protein overexpression in breast cancer is a risk factor in patients with involved and uninvolved lymph nodes. *Br. J. Cancer*, **63**, 434–438.

INTERNATIONAL UNION AGAINST CANCER (1987). *TNM Classification of Malignant Tumors*. 4th ed. Berlin, Springer Verlag.

KAPLAN, E.L. & MEIER, P. (1958). Non parametric estimation from incomplete observations. *J. Am. Stat. Assoc.*, **53**, 457–481.

LOVEKIN, C., ELLIS, I.O., LOCKER, A., ROBERTSON, J., BELL, J., GULLICK, W., ELSTON, C.W. & BLAMEY, R.W. (1989). C-erbB-2 oncogene expression in breast cancer: relationships and prognostic significance. *J. Pathol.*, **158**, 34b (Abstract).

MCGUIRE, W.L., TANDON, A.K., ALLRED, D.C., CHAMNESS, G.C. & CLARK, G.M. (1990). How to use prognostic factors in axillary node-negative breast cancer patients. *J. Natl Cancer Inst.*, **82**, 1006–1015.

NATIONAL INSTITUTE OF HEALTH (1986). Proceedings of the NIH Consensus Development Conference on Adjuvant Chemotherapy and Endocrine Therapy for Breast Cancer, NCI *Monograph nr.*, 1, Bethesda, National Cancer Institute.

O'REILLY, S.M., BARNES, D.M., CAMPLEJOHN, R.S., BARTKOVA, J., GREGORY, W.M. & RICHARDS, M.A. (1991). The relationship between c-erbB-2 expression, S-phase fraction and prognosis in breast cancer. *Br. J. Cancer*, **63**, 444.

PAIK, S., HAZAN, R., FISHER, E.R., SASS, R.E., FISHER, B., REDMOND, C., SCHLESSINGER, J., LIPPMANN, M.E. & KING, C.R. (1990). Pathologic findings from the National Surgical Adjuvant Breast and Bowel Project: Prognostic significance of C-erb B-2 protein over expression in primary breast cancer. *J. Clin. Oncol.*, **8**, 103–112.

PERREN, T.J. (1991). C-erbB-2 oncogene as a prognostic marker in breast cancer. *Br. J. Cancer*, **63**, 328–332.

QUERZOLI, P., MARCHETTI, E., FABRIS, G., MARZOLA, A., FERRETTI, S., IACOBELLI, S., HAZAN, R., RICHTER KING, C. & NENCI, I. (1990). Immunohistochemical expression of c-erbB-2 in human breast cancer by monoclonal antibody: correlation with lymphnode and ER status. *Tumori*, **76**, 461–464.

SCHECHTER, A.L., STERN, D.F., VAIDYANATHAN, L., DECKER, S.J., DREBIN, J.A., GREENE, M.I. & WEINBERG, R.A. (1984). The neu oncogene: an erb-B-related gene encoding a 185,000-Mr tumour antigen. *Nature*, **312**, 513–516.

SLAMON, D.J., CLARK, G.M., WONG, S.G., LEVIN, W.J., ULLRICH, A. & MCGUIRE, W.L. (1987). Human breast cancer: correlation of relapse and survival with amplification of the HER-2/neu oncogene. *Science*, **235**, 177–182.

SOOMRO, S., SHOUSHA, S., TAYLOR, S., SHEPARD, H.M. & FELDMANN, M. (1991). C-erbB-2 expression in different histological types of invasive breast carcinoma. *J. Clin. Pathol.*, **44**, 211–214.

TANDON, A.K., CLARK, G.M., CHAMNESS, G.C., ULLRICH, A. & MCGUIRE, W.L. (1989). HER-2/neu oncogene protein and prognosis in breast cancer. *J. Clin. Oncol.*, **7**, 1120–1128.

VAN DE VIJER, M.J., PETERSE, J.I., MOOI, W.J., WISMAN, P., LOMANS, J., DALESIO, O. & NUSSE, R. (1988). Neu-protein overexpression in breast cancer. Association with comedo-type ductal carcinoma in situ and limited prognostic value in stage II breast cancer. *N. Engl. J. Med.*, **319**, 1239–1245.

VARLEY, J.M., SWALLOW, J.H., BRAMMAR, W.J., WHITTAKER, J.L. & WALKER, R.A. (1987). Alterations to either c-erbB-2 (neu) or c-myc proto-oncogenes in breast carcinomas correlate with poor short term prognosis. *Oncogene*, **1**, 423–430.